# Recycled human hair-derived activated carbon for energy-related applications

**DOI:** 10.3906/kim-2105-70

**Published:** 2021-10-05

**Authors:** Oğuzhan KOTAN, Hatice BAYRAKÇEKEN

**Affiliations:** Department of Chemical Engineering, Faculty of Engineering, Atatürk University, Erzurum, Turkey

**Keywords:** Biomass, human hair, activated carbon, BET surface area, supercapacitor

## Abstract

Activated carbon, having high surface area and porosity, is a carbonaceous material that comes from organic main sources. In this work, activated carbon materials were produced from human hair that belongs to people who can be classified into two age groups (0–18 or 18–40 years). Activated carbons were characterized by nitrogen adsorption/desorption isotherms (BET), X-ray diffraction (XRD), Fourier Transform Infrared Spectroscopy (FTIR), Raman spectroscopy, scanning electron microscopy (SEM), and elemental analysis techniques. Capacitances of the synthesized materials were determined by using cyclic voltammetry (CV). Characterization results showed that the structural properties of activated carbon materials and capacitance values are changed significantly according to different age groups. BET surface areas of the 0–18 and 18–40 age groups were obtained as 2303 m^2^/g and 2674 m^2^/g, respectively. It was observed that 18–40 age group showed higher specific capacitance (294 F/g) than the 0–18 age group (219 F/g) due to high surface area.

## 1. Introduction

Activated carbon, having a high surface area and porosity, is a carbonaceous material. It is derived from the carbon-rich organic precursors such as coal, polymer, or biomass having higher carbon content via physical or chemical activation of these materials at high temperatures in order to increase the carbon content [1]. In other words, activated carbon is obtained by thermal decomposition of the carbon-rich organic materials having higher carbon content. It is well defined in the literature that activated carbon is obtained by physical or chemical activation of the carbon-rich organic materials[2]. Briefly, physical activation can be carried out via single stage [3] or two [4] stage processes. In the commonly used two stage process, carbonization of the carbon-rich material is achieved in a reactor in inert atmosphere, and then activation is occurred by using CO_2_, steam, air, or their mixtures in order to increase the surface area and porosity [5]. Chemical activation process is a single stage process in which the carbonaceous material is mixed with the activating agents such as potassium hydroxide, phosphoric acid, and zinc chloride, and then activated carbon is obtained by applying high temperatures under inert atmosphere [1]. It is aimed to synthesize high surface area and highly porous activated carbon materials by using either activation processes.

Activated carbon is used in a considerable number of different sectors such as gas masks, gas purification, gold purification, water purification, pharmaceutical, sewage treatment, and respirator and air filters, and other various applications [6]. Activated carbon is another form of carbon processed to have unique properties such as tunable porosity, lightweight, electrical conductivity, chemical inertness, etc. due to the wide range of morphologies and these properties it is used as supercapacitor material [7,8]. It is important to control parameters such as raw material, surface area and pore size distribution, extent of graphitization, heteroatom content, surface concentration of heteroatoms, type of functional groups, activation time, activation temperature during the preparation of activated carbon [9]. In addition to substances which contains high cellulose such as peanut shells, coconut shells, woods, walnuts, activated carbon is also obtained from carbonated sources including peat, lignite, coal, and petroleum. Particularly waste biomass can contribute to produce activated carbon [10]. In this respect, waste hair, considered as the main ingredient for activated carbon, will contribute to the economy. Human hair constitutes one of the most significant biological waste, which is destroyed by means of being burning in cities. Because of this combustion process, it leads to the emotion of foul-smelling and irritating substances including sulphide compounds and ammonia [11].

Waste hair includes many applications in industry and academic research. Human hair is composed of chemically excellent chemical composition and several interesting features. Due to these properties, the use of waste human hair for different applications is very attractive. Human hair is composed of approximately 91% polypeptides including more than 50 % carbon and the remaining parts consist of elements such as oxygen, hydrogen, nitrogen, and sulphur [12]. Hair consists of three main parts: cuticle, cortex, and medulla, which are formed via the keratinization of dying cells growing out of the root [13]. The main component of human hair is the keratin protein, which is comprised of 21 known amino acids. The studies related to the individual amino acids of keratin fibres mostly involve the amino acids cystine or tryptophan [14]. There are some studies in the literature that obtained activated carbon from human hair [15,16], but they did not specify the sex and age group the hair was taken from. There are several factors that affect the amino acid composition of human hair such as age [17], sex, hair colour, race, and also dietary habits and geographic origin [13]. The sex also has an impact on the amino acid composition. Human hair has been reported to contain more cysteine and cysteine in men’s hair than female hair, and dark hair contains more cysteine than light hair [18]. The effect of age on the distribution of tryptophan in human hair was also investigated, and it was reported that, like cysteine and cysteine results, tryptophan in male hair is significantly higher than in females. The content of tryptophan for both sexes was higher for dark hair than for light-coloured hair but reaches the highest level for grey and white hair, indicating that tryptophan accumulate between hair fibres with age. The authors also observed that colour did not affect tryptophan content at age intervals of 1–5 and 6–12 years, and the content of tryptophan considerably raised in women and men as their hair colour turn from light to dark between 20 and 40 years of age [19].

High power density, high efficiency, and long-life lead to considerable interest for supercapacitors, and they are divided into two types as electric double layer capacitors (EDLCs) and pseudocapacitors. However, EDCLs are better in terms of fast charge/ discharge rate, higher power density, higher columbic efficiency, and longer life when they are compared with pseudocapacitors [20]. Adsorption of electrolyte ions on high surface area conductive electrodes is the basics of EDLCs. Supercapacitor performance is strongly affected by the pore size distribution, surface area, surface functional groups, and degree of crystallinity of the carbon-based materials used as EDLC electrodes [21].

Activated carbon which is obtained from hair is used as a supercapacitor because of its high surface area and high porosity. The surface areas of the supercapacitor electrode materials are less than 2000 m^2^/g. In the literature, there are some studies related with the supercapacitor electrode material preparation from human hair. Porous carbon flakes added with heteroatom were obtained by carbonization of human hair in which the specific capacitance was achieved at 340 F/g in a 6 M KOH solution [22]. Activated carbon (AC)-MnO_2_ composite was used as supercapacitor electrode material and MnO_2_/ACs (1:12) achieved a maximum capacitance of 410 F/g, 345 F/g and 291 F/g in 1.0 M H_2_SO_4_, 1.0 M KOH, and 1.0 M Na_2_SO_4_ electrolytes, respectively [23].

The main objective of this study is originated to search for the effect of different age groups (since aging provides structural changes in human hair) on the properties of human hair sourced activated carbon and used them as supercapacitor material. In this study, untreated dark colour male human hair from various age groups 0–18 and 18–40 were used in order to synthesize activated carbon materials for possible utilization as supercapacitor electrode materials. To the best of our knowledge, this is the first-time human hair at different age groups derived activated carbon materials for supercapacitor applications is reported in the literature. Activated carbon materials were then physically and electrochemically characterized.

## 2. Materials and methods

### 2.1. Synthesis of activated carbon

In the current study, dark-colored male human hair was used to produce activated carbon. For this study, men’s hair was preferred because men’s hair is not usually dyed, and it is not exposed to chemical treatment. It was divided into two groups, ages 0–18 and 18–40 years old. Prior to the experiment process, isopropanol was used to wash hair samples, and these samples were fired in an oven at 80 °C. These samples were washed with isopropanol, cut about 5 mm in length, and carbonized in the oven under argon atmosphere at 300 °C for 90 min. Following that process, charred materials were mixed with KOH and carbonized at 800 °C for 120 min under argon atmosphere. In the literature, activators such as H_3_PO_4_, ZnCl_2_ and KOH have been used either alone or as a mixture of them for the activation of human hair. However, as a result of experimental data, it was determined that H_3_PO_4_ and ZnCl_2_ cause pore shrinkage. However, KOH showed the best electrochemical performance among these activators [24]. These final materials were mixed with 1M HCl solution, and then they were thoroughly washed with distilled water. The residues were collected and dried at 80 °C, and then activated carbon materials were obtained from human hair [21].

### 2.2. Physical characterization of activated carbon materials

Nitrogen adsorption/desorption isotherms were used to determine the structural properties of the activated carbon materials including surface area, pore size distribution and micropore volumes of the materials. Micromeritics 3Flex Surface Characterization Analyzer is used to determine the structural properties. X-ray diffraction (XRD) analysis was used to determine the crystal structure of the activated carbon materials. XRD analysis is conducted with PANalytical Empyrean X-Ray Diffractometer by using Cu Kα radiation source operating at 45 kV and 40 mA over 2Θ range of 10°–90°. Fourier Transform Infrared Spectroscopy (FTIR) was used to determine the surface functional groups of the activated carbon materials obtained from different age groups. FTIR analysis is conducted by using a VERTEX 80v FTIR spectrometer. The surface morphology of the activated carbon materials was determined by using scanning electron microscope (SEM) via Zeiss Sigma 300 Field Emission device. The Raman spectra of the activated carbon materials were obtained by using a Micro Raman (WITech alpha 300R). Elemental analysis of the raw human hair samples and also activated carbon materials were obtained by LECO CHNS932 instrument.

### 2.3. Electrochemical characterization of activated carbon materials

Capacitances of the activated carbon materials were determined by using cyclic voltammetry (CV) technique. All experiments were carried out at ambient temperature by using a standard three electrode cell configuration. Glassy carbon (GC), Ag/AgCl, and platinum wire electrodes were used as working, reference and counter electrodes, respectively. Required amounts of the activated carbon materials of different age groups were mixed with deionized water, 1,2 propandiol and 20% Nafion solution. After ultrasonication of this mixture, 2 Ml is taken and put onto the GC electrode. The activated carbon material amount was set to 28 μg/cm^2^ for all CV experiments. Cyclic voltammograms were recorded in 1 M H_2_SO_4_ electrolyte. Before passing through the measurements, the electrolyte solution was purged with nitrogen for half an hour to eliminate the dissolved oxygen from the electrolyte solution. Cyclic voltammograms were recorded after 50 cycles for a scan rate of 50 mV/s.

## 3. Results and discussion

**Figure 1:**
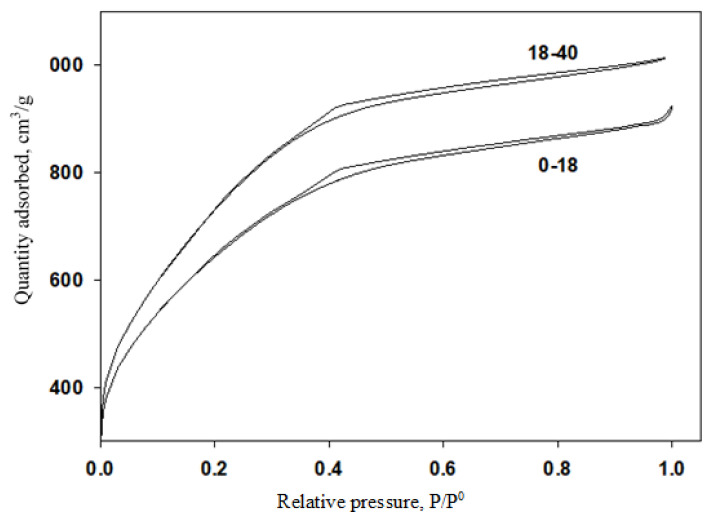
Nitrogen adsorption/desorption isotherms for the activated carbon materials.

**Figure 2:**
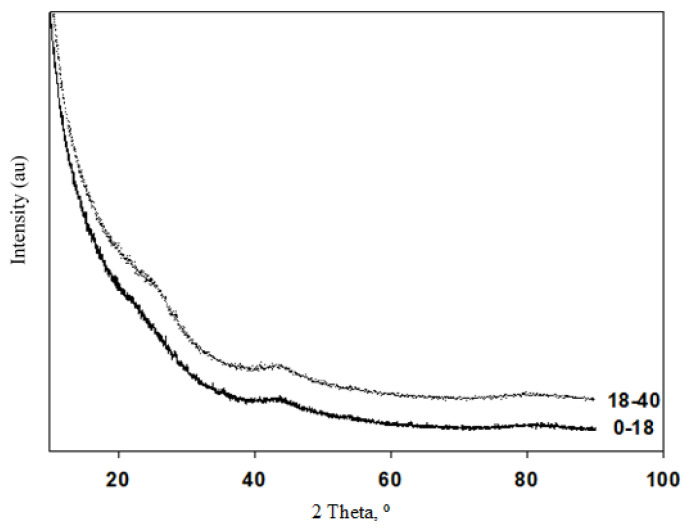
X-Ray Diffraction (XRD) patterns of the synthesized activated carbon materials.

The nitrogen adsorption/desorption isotherms for the activated carbon materials are given in Figure 1. Both activated carbon materials exhibited Type IV adsorption/desorption isotherms with H4 hysteresis loops according to IUPAC classification. This trend can be attributed to the microporous and mesoporous structures with slit-like micropores interconnecting with mesopores [25]. The adsorption-desorption hysteresis loops for both materials were obtained at P/P^0^ values in between 0.4 and unity indicating the mesoporosity of the materials [26]. The structural properties of the activated carbon materials obtained from human hair of different age groups are tabulated in Table 1. As seen from the table that the surface area of 18–40 age group was found to have higher surface area than the one obtained from 0–18 age group. The comparison of the two groups showed that the total pore volume and micro pore volume of activated carbon, which is obtained from the older age, were higher than the one in young age group. The increase in surface area and total pore volume may be due to the effect of the increase in micropores. A slight decrease in pore diameter was observed for an increase in age group. These changes can be attributed to the compositional changes in the human hair by age [17,19]. Figure 2 shows the XRD plots for activated carbon materials obtained for groups divided by age. As can be seen from the evaluation of these graphics, amorphous structures were obtained for the synthesized materials. Also, the graphical stacking peak, which is around 23° for the 18–40 age group, is clearly visible. Similarly, a peak at 43.8 °C was clearly seen for 0–18 and 18–40 age groups. The peaks located at 23° and 43.8° can be attributed to the (002) and (101) planes of the hexagonal graphite. High density of micropores were confirmed with the intensity increase at low-angle scattering peaks [22].

**Figure 3:**
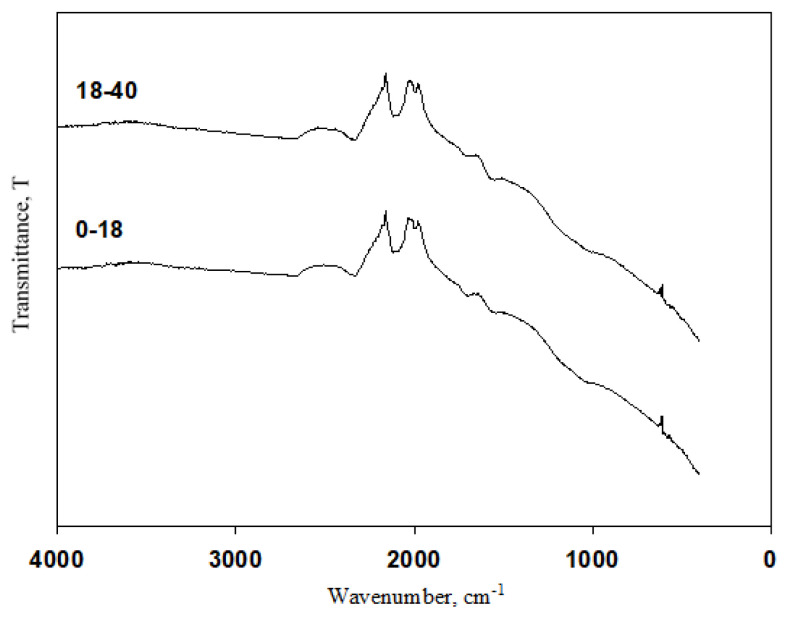
Fourier Transform Infrared Spectroscopy (FTIR) results of the synthesized activated carbon materials.

Surface functional groups of the activated carbon materials were determined by using FTIR spectra. Figure 3 shows the FTIR results of synthesized activated carbon materials. For activated carbon from both age groups, similar functional groups were obtained. The peaks at 1710 and 1562 cm^−1^ represent the C = O bonds of the carboxylic groups (= COOH) and the tensile vibrations of the conjugated C-C bonds of the aromatic rings [27]. The peaks at 1707 and 1580 cm^−1^ represent C=O bonds of the carboxylic groups (–COOH) and the stretching vibrations of conjugated C–C bonds of aromatic rings, respectively [28]. The peaks located at 2325 cm^−1^ is the C≡ C stretching vibrations in alkyne groups [29]. Peaks located in the range of 2364–2372 cm^−1^ can be attributed to C-H stretching due to presence of CH_2_–CO groups [30]. Similar trends were observed for each activated carbon materials obtained for both age groups.

**Figure 4:**
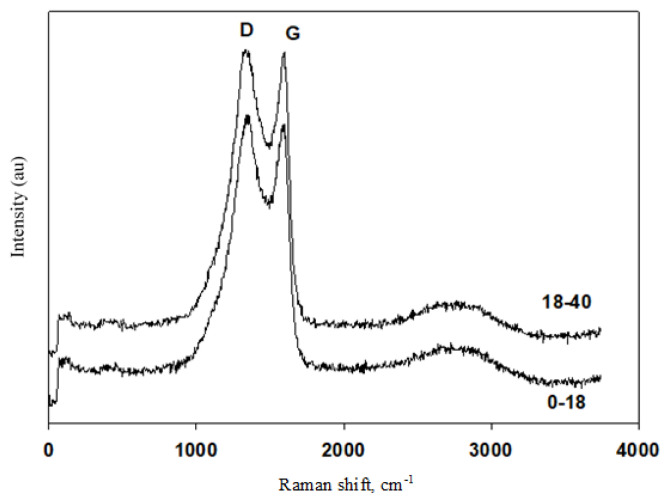
Raman spectra of the activated carbon materials.

**Figure 5:**
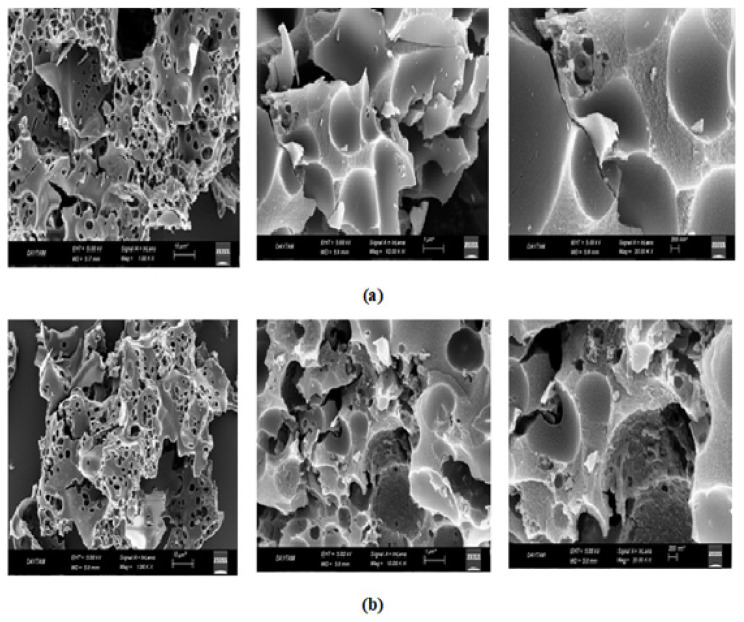
Scanning Electron Microscope (SEM) images of the synthesized activated carbon materials (a) 0–18 (b) 18–40.

Raman spectra of the activated carbon materials are given in Figure 4. Activated carbon of 0–18 age group has the D and G bands values of 1344 and 1571 cm^−1^, and 18–40 age group has the D and G bands values of 1332 and 1583 cm^−1^. D band represents the defects of the carbon materials, whereas G band is attributed to graphitic characteristic. The ratio of D band to G band (ID/IG) shows the structural defect degree of the material according to graphitic structure. The ratio of D band to G band (ID/IG) values were obtained as 1.01 and 1.00 for the activated carbon materials obtained for 0–18 and 18–40 age groups, respectively, which indicates that the activated material in 18–40 age group has slightly more graphitic structure. In literature, it was shown that the hollow carbon fibers obtained from human hair have an ID/IG ratio of 0.99, and it is mostly glassy carbon [12]. In another study, it was also shown that carbon flake obtained from human hair have an ID/IG ratio of 1.1, which is suitable for utilization in supercapacitors [22]. SEM images for the synthesized activated carbons are given in Figure 5. Dense structures with holes were obtained for both activated carbon materials. Smaller holes with more porous structure are obtained for the activated carbon synthesized with 18–40 age group, which also contribute to high surface area of this material. These visible pores can be attributed to the addition of KOH during the synthesis of the activated carbon materials. Existence of KOH positively affects the pore formation by eliminating volatile elements during chemical activation [31]. This enhancement can be attributed to the following reaction proposed by Chayid and Ahmed, 2015:


$$6\text{KOH}+2\text{C}\to 2\text{K}+3{\text{H}}_{2}+2{\text{K}}_{2}{\text{CO}}_{3}$$

**Figure 6:**
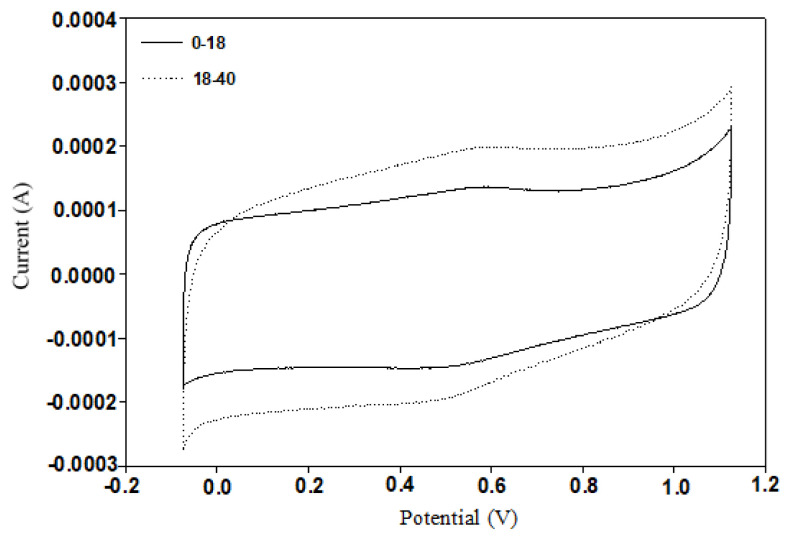
Cyclic voltammograms of the synthesized activated carbon materials.

Subsequently, the product K_2_CO_3_ was either reacted with carbon to produce K and CO or decomposed to K_2_O and CO_2_ in order to produce extra pores. SEM images showed the existence of cavities on the activated carbon materials surfaces that were formed due to the emission of gaseous products [32]. These cavities served as the access points of the adsorbate molecules into the internal pores of the carbon particle [31]. Elemental compositions of the activated carbon materials can be determined by using different analysis techniques including XPS, EDS, etc. In this study, elemental analysis method is used to determine the elemental composition of the corresponding activated carbon materials. Elemental analysis results for raw human hair samples and also the activated carbon materials are given in Table 2. It was clearly seen that the carbon ratio of the activated carbon materials is increasing and hydrogen, nitrogen, and sulphur amounts are decreasing when compared to the raw human hair samples. It is an expected result after pyrolysis process. Carbon ratio of 18–40 age group was higher than the 0–18 age group before and after the pyrolysis process, and the difference increased after pyrolysis. In the literature, it was observed that, with EDS data, the human hair has the elemental compositions of carbon (51.19 %), oxygen (27.46%), nitrogen (17.55%), sulphur (3.06%), and, after KOH activation, the carbon content increased to 83.88%, and the nitrogen content was reduced to 5.53% [33]. It was also observed that the carbon contents of both activated carbon materials increased when compared to the raw human hair sources at different age groups. The specific capacitances of the activated carbon materials can be calculated by using the following equations:


(1) $$\text{C}=\frac{Q}{V}$$

(2) $$\text{C}=\frac{\int \text{idV}}{2\text{Vs }\Delta \text{V}}$$

(3) $${\text{C}}_{\text{s}}=\frac{C}{m}$$

which include the ratio of the electrical charge (Q) to the potential difference (V) between each electrode [34] where ∫idV is obtained by the integration of the area under the CV curve, V_s_ the potential scan rate, and ΔV the potential range. Specific capacitance (Cs) can be obtained by dividing the capacitance by the mass of the active material (m) [35]. The specific capacitances of the activated carbon materials are calculated by using cyclic voltammograms, and the equations given above. Corresponding cyclic voltammograms are given in Figure 6. Specific capacitances of the activated carbon materials were obtained as 294 and 219 F/g for 18–40 and 0–18 age groups, respectively. It was observed that 18–40 age group showed higher specific capacitance than 0–18 age group due to its high surface area. This situation can be attributed to the difference between the micropore structures of the synthesized materials which affect the electrolyte to reach the active surface area. Specific capacitances obtained from the human hair sourced activated carbon based materials from this study and also the literature via three electrode cell configuration are summarized in Table 3. As can be seen from table, there are significant differences in the structural properties and also the capacitance values of the activated carbon materials sourced from human hair depending on the electrolyte used and utilization of AC with other metal oxides.

## 4. Conclusion

Human hair as a common and general waste will be available in the world, and it is of considerable significance to evaluate this continuous source. Thus, disposing this waste in useful materials is of great importance. In the current study, human hair from various age groups (0–18 and 18–40) is used to synthesize activated carbon materials. It was observed that the obtained surface areas of the materials show variation depending on the age. The variation between structural changes in activated carbons resulted in a significant change in specific capacitance values. In the current study, it has been stated that the features of activated carbon vary according to the age group the hair obtained due to the changes occurring in the structures of the human hair by aging.

## Figures and Tables

**Table 1:** Structural properties of the activated carbon materials.

Material	BET surface area, m^2^/g	BJH average total pore volume, cm^3^/g	Micropore volume, cm^3^/g	Average pore diameter, nm
**0–18**	2303	1.0309	0.0251	2.38
**18–40**	2674	1.2371	0.0983	2.31

**Table 2:** Elemental analysis results for raw human hair samples and the activated carbon materials.

	C (%)	H (%)	N (%)	S (%)
**H018**	45.25	6.532	14.89	4.53
**H1840**	46.04	6.582	15.24	4.663
**018AC**	68.79	1.370	3.826	1.071
**1840AC**	72.02	1.222	4.556	0.756

**Table 3:** Capacitance values of activated carbons obtained from human hair according to different electrodes.

Material	Electrolyte	BET surface area (m^2^/g)	Capacitance (F/g)	Reference
AC from human (0–18)	1 M H_2_SO_4_	2303	219	In this study
AC from human hair (18–40)	1 M H_2_SO_4_	2674	294	In this study
AC from human hair (sex and age are not specified)	6 M KOH	1306	340	22
AC from human hair (sex and age are not specified)	1 M LiPF_6_ ethylene carbonate/diethyl carbonate (EC/DEC)	1306	126	22
MnO_2_/ACs (AC from human hair, sex and age are not specified)	1.0 M H_2_SO_4_	1597.4	410	23
MnO_2_/ACs (AC from human hair, sex and age are not specified)	1.0 KOH	1597.4	345	23
MnO_2_/ACs (AC from human hair, sex and age are not specified)	1.0 Na_2_SO_4_	1597.4	291	23
